# Successful Treatment of a Coxofemoral Luxation in a Shetland Pony by Closed Reduction and Prolonged Immobilization Using a Full-Body Animal Rescue Sling

**DOI:** 10.1155/2020/2424653

**Published:** 2020-01-03

**Authors:** Miriam Sprick, Christoph Koch

**Affiliations:** Department of Clinical Veterinary Medicine, Swiss Institute for Equine Medicine (ISME), Vetsuisse Faculty, University of Bern and Agroscope, Bern, Switzerland

## Abstract

A 12-year-old, 170 kg, Shetland pony mare was presented with an acute severe right pelvic limb lameness and concurrent upward fixation of the right patella. The affected limb was rotated externally and adducted with a prominent greater trochanter and the right calcaneal tuber being more proximal than its left counterpart. Radiographic examination revealed complete dislocation of the right femoral head from the acetabular cavity in a dorsal and caudal direction. A closed reduction of the coxofemoral luxation was performed successfully under general anaesthesia. A full-body animal rescue and transportation sling (ARTS) was applied for the recovery. The reduction was followed by a right-sided medial patellar desmotomy. The pony was supported in the ARTS for a total of eight weeks combined with crossties for the first six weeks. Subsequently, the mare was discharged with instructions to slowly increase walking exercise over a period of two months before returning to her intended use. A follow-up after 22 months attested the successful treatment of a coxofemoral luxation by closed reduction and prolonged immobilization resulting in a regularly exercised pony without any residual lameness.

## 1. Introduction

Coxofemoral luxation is an uncommon injury in equids described mostly in young horses, miniature horses, and ponies [1–3]. The remarkably low prevalence in mature horses is attributed to a deep acetabulum, strong ligamentous support of the joint, including the equine exclusive accessory ligament, and heavy musculature that firmly stabilizes the coxofemoral joint [2, 4–6]. In adult horses, there is a higher chance of a fracture of the ileum than a luxation of the coxofemoral joint [7]. The most common cause of a coxofemoral luxation is trauma from a fall or a kick, but there are also reports of luxation secondary to limb immobilization in a full limb cast or to upward fixation of the patella [2, 4, 8].

Surgical options for case management include open reduction followed by lateral stabilization, at least in small equids [6]. Since failure of this fixation has to be expected in equids with a bodyweight exceeding 150 kg, this can be combined with a toggle pin or prosthetic capsule technique [9]. Total hip arthroplasty is not an established treatment option in equids since it has only been described in a single case report, and a long-term follow-up could not be obtained because the pony succumbed to pulmonary fat embolism syndrome and small intestinal infarction following surgery [10]. Femoral head ostectomy can be performed as a last resort treatment but usually does not result in acceptable comfort except in very small equids [11–13].

Closed reduction is reported to have limited success in equids [1, 2, 4, 8, 14].

To the authors' best knowledge, only a small number of reports on successful closed reduction of the coxofemoral luxation in equids can be found in the veterinary literature. These include one Quarter horse filly with an unknown long-term outcome and two Shetland ponies with a good long-term outcome, followed for up to 2 and 3 years, respectively [2, 4, 14]. However, both Shetland ponies were reported to have a noticeable residual lameness following the coxofemoral luxation [2, 3]. The aim of this report was to describe the successful management and long-term outcome of a 170 kg Shetland pony mare following correction of coxofemoral luxation by closed reduction.

## 2. Case History

A 12-year-old, 170 kg, Shetland pony mare was presented to the emergency service of the ISME Equine Clinic Berne for evaluation of an acute severe right pelvic limb lameness. The mare was used for pleasure riding by children. There was a history of laminitis, and the owner reported episodes of intermittent upward fixation of the right patella. The pony was found lame on pasture on the day of admission.

## 3. Clinical Findings

Upon presentation, the mare was in a good general condition and showed a severe right pelvic limb lameness (Grade 4 of 5 according to the scale of the American Association of Equine Practitioners) with concurrent upward fixation of the right patella. External rotation and adduction of the right pelvic limb were observed with the right greater trochanter more prominent and the right calcaneal tuber being more proximal than its left counterpart. Physical examination of the mare was normal, and no wounds could be detected. Based on these findings, the differential diagnoses were a coxofemoral luxation or pelvic fracture involving the right acetabulum, and radiographs of the right coxofemoral joint were obtained. The mare was sedated with romifidine (0.04 mg/kg intravenously (IV)) and L-methadone (0.05 mg/kg IV). General anaesthesia was induced with ketamine (2.5 mg/kg IV) and diazepam (0.05 mg/kg IV), and anaesthesia was maintained with isoflurane in oxygen and a continuous rate infusion of romifidine (0.04 mg/kg/h IV). Radiographic examination of the pelvis was subsequently performed with the pony in dorsal recumbency.

## 4. Diagnosis

**Figure 1:**
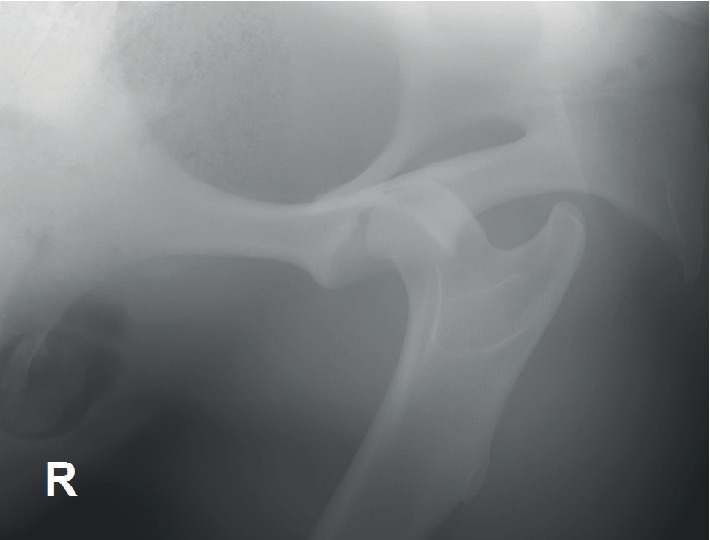
Ventrodorsal radiographic projection of the right acetabulum with complete dislocation of the right femoral head in a dorsal and caudal direction.

A laterolateral and ventrodorsal radiographic projection centred over the right acetabulum revealed complete dislocation of the right femoral head from the acetabular cavity in a dorsal and caudal direction (Figure 1).

## 5. Treatment

**Figure 2:**
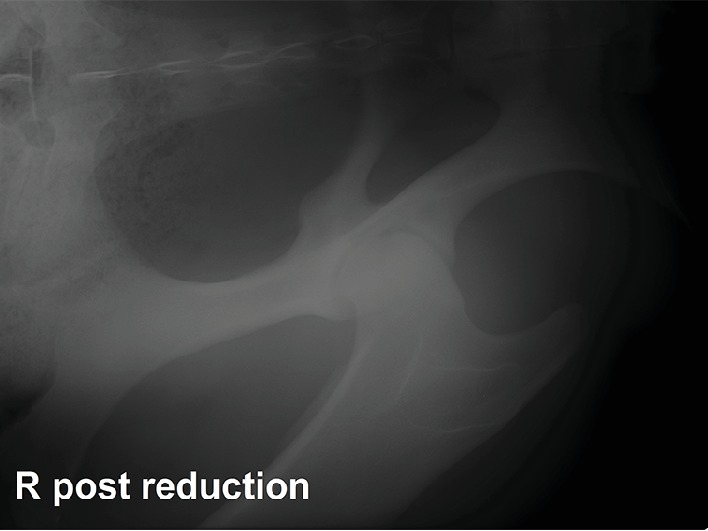
Ventrodorsal radiographic projection of the right acetabulum after closed reduction of the coxofemoral luxation. The right femoral head is seated in its correct position within the acetabulum.

Closed reduction of the coxofemoral luxation was performed while the mare was still anaesthetised. To achieve this, the pony was left in dorsal recumbency and a rope was placed around the pastern of the right pelvic limb to apply distal traction on the extremity using a hoist. After several attempts, traction combined with external rotation and adduction by the surgeon allowed a reduction of the femoral head back into the acetabular cavity. Reduction was completed by internal rotation of the limb. Laterolateral and ventrodorsal radiographs confirmed the complete reduction (Figure 2).

**Figure 3:**
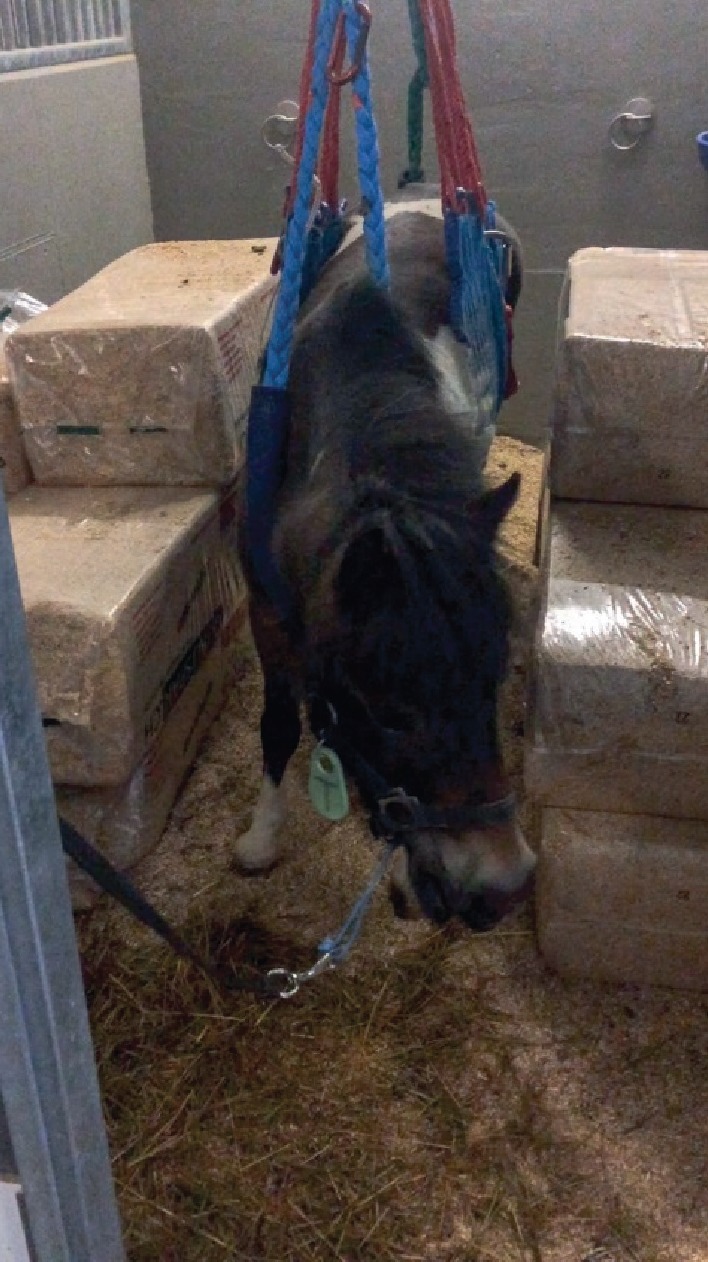
A crosstied Shetland pony mare in a full-body animal rescue and transportation sling (ARTS) after closed reduction of a right coxofemoral luxation.

A full-body animal rescue and transportation sling (ARTS) was applied for the recovery [15]. Additionally, an Ehmer sling was placed on the right pelvic limb for the recovery period and for the first 12 hours following closed reduction. The following day, the Ehmer sling was removed, due to a lack of compliance for the sling by the pony, but the pony was maintained in the full-body ARTS. Furthermore, the pony was crosstied, and a couloir of approximately 5 feet width was made with large bales of wood shavings to keep the pony from moving from side to side with its rear end (Figure 3). Within hours after removing the Ehmer sling, the pony showed an upward fixation of the right patella. Therefore, a right-sided medial patellar ligament desmotomy was performed under local anaesthesia and sedation.

## 6. Further Case Management

Nonsteroidal anti-inflammatory drugs, flunixin meglumine (1.1 mg/kg IV, once daily) for four consecutive days, and meloxicam (0.6 mg/kg orally, once daily) were administered for an additional five days. Analgesia was continued with firocoxib (0.1 mg/kg orally, once daily) for three additional weeks. The pony was supported in the ARTS net and crosstied for six weeks. After these six weeks, the bales of wood shavings and crossties were removed, and the mare was allowed to move around freely in the box. However, the mare was kept in the ARTS to prevent her from lying down. Because intermittent left-sided upward fixation of the patella had been observed, a left-sided medial patellar ligament desmotomy was performed under local anaesthesia and sedation.

Starting seven weeks after closed reduction, the pony was walked out of the box stall and allowed to hand-graze daily. After a total of eight weeks, the ARTS was removed and the pony was allowed to lie down and move freely in the box stall. In week nine, after observing that the pony had laid down and gotten back up on its feet without complications, the pony was discharged from the hospital with instructions for continued box rest and progressively increasing daily hand walking for up to 30 minutes per day for the following two months.

## 7. Outcome

The owner and referring veterinarian provided weekly updates and videos of the pony. No complications were reported in the two months following hospital discharge, and the pony was returned to regular pasture turnout four months after the closed reduction. By six months after hospital discharge, the pony was used again for pleasure riding by young children without any signs of residual lameness, as assessed on video footage and on site by the referring veterinarian. The last follow-up information was obtained just prior to submission of this case report, 22 months following closed reduction of the coxofemoral joint luxation, and the mare was still used regularly as a riding pony for children without any observable residual lameness.

## 8. Discussion

In select cases, coxofemoral luxation can be successfully treated by closed reduction and adequate immobilization in the weeks following the closed reduction. A full-body animal rescue and transportation sling and crosstying were well-tolerated means of constraint to achieve adequate postoperative immobilization in the described case.

Both the accessory ligament and the smaller ligament *capitis ossis femoris* need to rupture for a luxation to occur [7]. Interestingly, coxofemoral luxation may also be associated with upward fixation of the patella, especially in ponies and miniature horses [2, 4, 16], and speculated to occur secondary to violent *quadriceps femoris* contractions caused by forceful attempts to flex the limb locked in extension [4]. Alternatively, upward fixation of the patella can occur secondary to coxofemoral luxation when rotation of the limb impedes the function of the *rectus* and *biceps femoris* muscles in the so-called patellar release mechanism [16]. Based on this information, it can be argued that medial patellar desmotomy is an effective, adjunctive treatment for coxofemoral luxation and should be routinely performed immediately after open or closed reduction of a coxofemoral luxation to reduce the risk of reluxation, especially in animals that have a history of previous upward fixation of the patella. In the present case, the upper fixation of the patella was released following closed reduction of the coxofemoral luxation but reoccurred immediately after removing the Ehmer sling the following day.

A complete diagnostic radiographic evaluation of the coxofemoral joint in dorsal recumbency under general anaesthesia is recommended to confirm luxation [7, 17]. However, dynamic ultrasonography or standing lateral oblique radiographs of the pelvis, alone or in combination, can provide diagnostic images when assessing a suspected coxofemoral luxation in small equids [18–20]. In the present case, general anaesthesia for radiographic assessment of the highly suspected luxation was preferred so that immediate closed reduction could be performed. Nonetheless, standing ultrasonography was performed successfully and repeatedly in the present case after closed reduction, to assess the position of the femoral head and to rule out a reluxation, whenever the pony was reluctant to bear full weight on the affected limb.

Closed reduction of coxofemoral luxation is rarely successful and is commonly associated with a high incidence of reluxation (up to 80%) [1, 2, 4, 8]. However, three isolated cases of successful closed reduction have been reported in the veterinary literature [2, 4, 14]. The likely causes of the high rate of reluxation are accumulation of blood clots and debris within the acetabular socket, damage to the capsular muscles, and trapping of the fibrocartilage rim within the acetabulum preventing proper seating of the femoral head [6, 21, 22]. Based on experiences in cattle, closed reduction is more likely to be successful if performed within 36 hours after luxation. Once this time window has passed, closed reduction will likely be difficult to achieve, because of muscle contraction and the formation of organised blood clots within the acetabulum [21, 22]. Therefore, a short time frame between initial injury and closed reduction, as in the present case, seems to be an important factor for success.

A controlled and uneventful recovery from general anaesthesia and adequate immobilization during the postreduction period are critical factors for a successful outcome. Reluxation after closed reduction is described to occur in 80% of the cases during recovery from general anaesthesia or in the ensuing 3 days [2]. This was also the case in the first attempt of closed reduction in a Shetland pony published in the English veterinary literature [4]. In this original report, Clegg and coworkers applied an Ehmer sling during recovery and the first 4 days postoperatively. Likewise, an Ehmer sling was used in the case presented here, but the use of the sling was restricted to the first 12 hours following general anaesthesia. The Ehmer sling subsequently was removed because the pony was constantly trying to free the affected limb from the restrictive bandage. Besides the lack of compliance, as experienced in this case, another potential disadvantage associated with placing an Ehmer sling is the increased risk of support limb laminitis.

We speculate that preventing the pony from lying down during the 2 months following reduction and avoiding sudden forces to act on the capsule of the affected coxofemoral joint were not only essential in preventing reluxation but also allowed for a functional healing of the traumatized joint capsule. The combined use of the ARTS and crossties on the pony in a well-confined space proved to be an effective method of immobilization, and the pony tolerated it well. The fortunate combination of timely corrective intervention and effective postreduction management allowed this pony to return to its prior function without any residual lameness.

## 9. Conclusion

Closed reduction under general anaesthesia followed by medial patellar desmotomy and two months of immobilization using a commercially available ARTS combined with crossties can be a valid treatment approach for the treatment of craniodorsal coxofemoral luxation in mature equids weighing less than 200 kg. Provided that the collateral damage to the coxofemoral articulation is limited and a prompt and properly executed closed reduction is performed, a full return to previous use is possible.
